# Sex Morality

**Published:** 1915-05

**Authors:** Malone Duggan


### Book Notices.

SEX MORALITY.—Past, Present and Future. By Wm. J. Robinson, M. D., and others. Published by The Critic and Guide Co. Price, $1.00.

A confrere of mine once said, in discussing skin diseases, that all skin diseases could be grouped into two classes: One class are those which K. I. will cure and the other are those which all H—— cannot cure. This laconic expression, while very brusque, is strikingly applicable to the attitude people take toward the question of sex. Some people, freed from the bondage of atavistic and inherent Puritanic conceptions of sex morality, are open to conviction and the dispassionate discussion of the subject. And there are those so affected with this social and hereditary propensity that no amount at coaxing or argument could make them see differently. To the former class this book of Dr. Robinson's will appeal. It discusses unbiasedly and openly, in succinct and eloquent terms, the questions of continence and incontinence, prenuptial and extra-marital relations and their bearings on health and morals. Seven distinct viewpoints are given from as many different authors. And, while their opinions differ, the combined information affords a clear prospectus of the whole question and a richness of information that is not easily found in literature. The relation of the sexes is traced from the early dawn of the simple life to the present complicated system of ethics. The moral customs of many of the savage and civilized races are recounted and the customs of the future are forecast.

The little volume can be read in an evening, and will give thought for deep consideration for many evenings. Any attempt towards a review of the book would lead into a discussion beyond the limits of this space, and, after all, would not come so near the point as the personal perusal of the book itself.

MALONE DUGGAN, M. D.

